# Reduced grey matter volume in adolescents with conduct disorder: a region-of-interest analysis using multivariate generalized linear modeling

**DOI:** 10.1007/s44192-023-00052-3

**Published:** 2023-11-17

**Authors:** Ru Zhang, R. James R. Blair, Karina S. Blair, Matthew Dobbertin, Jaimie Elowsky, Johannah Bashford-Largo, Ahria J. Dominguez, Melissa Hatch, Sahil Bajaj

**Affiliations:** 1 Mark and Mary Stevens Neuroimaging and Informatics Institute, Keck School of Medicine, University of Southern California, Los Angeles, CA 90033 USA; 2 Child and Adolescent Mental Health Centre, Mental Health Services, Capital Region of Denmark, Copenhagen, Denmark; 3 Center for Neurobehavioral Research, Boys Town National Research Hospital, Boys Town, NE USA; 4 Inpatient Psychiatric Care Unit, Boys Town National Research Hospital, Boys Town, NE USA; 5 Department of Psychology, University of Nebraska-Lincoln, Lincoln, NE USA; 6 Center for Brain, Biology, and Behavior, University of Nebraska-Lincoln, Lincoln, NE USA; 7 Clinical Health, Emotion, and Neuroscience (CHEN) Laboratory, Department of Neurological Sciences, College of Medicine, University of Nebraska Medical Center (UNMC), Omaha, NE USA; 8 Mind and Brain Health Labs (MBHL), Department of Neurological Sciences, College of Medicine, University of Nebraska Medical Center (UNMC), Omaha, NE USA; 9 Department of Cancer Systems Imaging, Division of Diagnostic Imaging, The University of Texas MD Anderson Cancer Center, Houston, TX USA

## Abstract

**Background:**

Conduct disorder (CD) involves a group of behavioral and emotional problems that usually begins during childhood or adolescence. Structural brain alterations have been observed in CD, including the amygdala, insula, ventrolateral and medial prefrontal cortex, anterior cingulate cortex, and fusiform gyrus. The current study developed a multivariate generalized linear model (GLM) to differentiate adolescents with CD from typically developing (TD) adolescents in terms of grey matter volume (GMV).

**Methods:**

The whole‐brain structural MRI data were collected from 96 adolescents with CD (mean age = $16.188\pm 1.259$ years; mean IQ = $104.292\pm 8.107$; 63 males) and 90 TD individuals (mean age = $15.956\pm 1.506$ years; mean IQ = $106.622\pm 9.076$; 59 males) matched on age, IQ, and sex. Region-wise GMV was extracted following whole-brain parcellation into 68 cortical and 14 subcortical regions for each participant. A multivariate GLM was developed to predict the GMV of the pre-hypothesized regions-of-interest (ROIs) based on CD diagnosis, with intracranial volume, age, sex, and IQ serving as the covariate.

**Results:**

A diagnosis of CD was a significant predictor for GMV in the right pars orbitalis, right insula, right superior temporal gyrus, left fusiform gyrus, and left amygdala (*F*_(1, 180)_ = 5.460–10.317, *p* < 0.05, partial eta squared = 0.029–0.054). The CD participants had smaller GMV in these regions than the TD participants (M_CD_–M_TD_ = [− 614.898] mm^3^–[− 53.461] mm^3^).

**Conclusions:**

Altered GMV within specific regions may serve as a biomarker for the development of CD in adolescents. Clinical work can potentially target these biomarkers to treat adolescents with CD.

**Supplementary Information:**

The online version contains supplementary material available at 10.1007/s44192-023-00052-3.

## Introduction

Conduct disorder (CD) is a serious behavioral and emotional disorder that emerges in childhood and adolescence. It is characterized by a repetitive and persistent pattern of behavior in which the basic rights of others and/or major age-approximate social norms are violated [1]. CD is often seen as the precursor to adult antisocial personality disorder, thus has adverse long-term outcomes in both mental and physical domains of health [2]. CD is associated with an exceptionally high societal and economic burden, accounting for ~ 1% of all years lived with disability and surpassing autism spectrum disorders and attention-deficit/hyperactivity disorder (ADHD) in this measure of global health burden [3]. One significant critique of the diagnostic criteria for CD is that they solely rely on behavioral symptoms, providing little insight into the cognitive and emotional mechanisms that underlie these symptoms [4].

Recent functional neuroimaging studies have revealed that CD is associated with neurocognitive impairments in emotion processing [5], threat response [6, 7] and reinforcement-based decision-making [8–10]. The ventromedial prefrontal cortex (vmPFC), anterior cingulate cortex (ACC), insula, fusiform gyrus, temporal gyrus, and amygdala are particularly implicated as critical regions for the pathophysiology of CD. In addition, research has shown CD has structural abnormalities in the overlapping brain regions, suggesting that neural activity deficits may have a structural basis as well [8].

Previously, structural magnetic resonance imaging (sMRI) has been used to investigate the anatomical brain changes underlying CD, reflected by various morphometry parameters, including grey matter volume (GMV; i.e., cortical and subcortical volume [CV/SCV]). While a clear and consistent picture of the structural brain alterations seen in patients with CD remains lacking, two meta-analyses (each based on over 10 studies) of youth with CD, oppositional defiant disorder (ODD), or conduct problems concluded these conditions were associated with reductions in volume in cortical (ventrolateral, medial prefrontal, middle temporal, superior temporal, and anterior insular cortices) and subcortical (amygdala, caudate and putamen) regions [11, 12] though it should be noted that there have been several reports of both *increased* GMV in participants with CD relative to the controls in many of these regions since these meta-analyses were published [13–15] and a lack of group differences in GMV [16–19]. Within the pathology of CD, recent longitudinal studies suggested that higher CD-related symptomatology was associated with accelerated age-related cortical thinning [20, 21]. Specifically, Albaugh et al. [20] investigated a large community-based sample of 1039 adolescents. It revealed that a higher level of conduct problems (CP) was associated with reduced cortical thickness in late adolescence in the left rostral anterior cingulate, bilateral insula, and left inferior parietal cortices and this association was absent in early adolescence.

Moreover, there is a growing concern within the field of neuroimaging regarding replicability. For instance, MuGuire et al. [22] identified moderate-to-low reproducibility in sMRI when estimating gray matter thickness in several regions. Recent inquiries have also cast doubt on the replicability of structural brain-behavior associations [23, 24]. In developmental neuroimaging studies, maintaining consistency in repeated imaging has become increasingly challenging. This challenge arises from issues such as the difficulty in recruiting sufficiently large sample sizes within a limited age range and higher levels of in-scanner motion [25].

The current study primarily aimed to explore the structural brain alterations associated with CD using multivariate generalized linear modeling (GLM). We used the diagnosis of CD to predict the volumes of pre-hypothesized regions-of-interest (ROIs) taken from the meta-analysis by Rogers and De Brito [11]. The secondary aim of the current study was to evaluate if volume reduction in these ROIs reported by Rogers and De Brito [11] could be replicated. The ROIs we adopted were the right pars opercularis, right pars orbitalis, right pars triangularis, left superior frontal gyrus, right caudal anterior cingulate gyrus, bilateral insula, right superior temporal gyrus, left fusiform gyrus, and left amygdala (see Table 2, [11]). The diagnosis of CD was used as the predictor of the model, and intracranial volume, age, sex, and IQ served as the covariates. We hypothesized a significant association between the diagnosis of CD and reduced volume of the pre-hypothesized brain regions.

## Methods

### Participants

We included data from 186 young individuals aged ≥ 14 years (with IQ > 84; [26, 27]) from a residential care program and the surrounding community (mean age = $16.075\pm 1.385$ years; mean IQ = $105.419\pm 8.645$; 122 males). Youth recruited from the residential care program had been referred for behavioral and mental health problems. Participants from the community were recruited through flyers or social media. There were two groups of participants: participants with CD (*N* = 96; mean age = $16.188\pm 1.259$ years; mean IQ = $104.292\pm 8.107$; 63 males) and typically developing (TD) adolescents (*N* = 90; mean age = $15.956\pm 1.506$ years; mean IQ = $106.622\pm 9.076$; 59 males); see Table 1.

**Table 1:** Demographic and clinical variables

	Mean for CD	Mean for TD	*t* score	*p* value
Age	$16.188\pm 1.259$	$15.956\pm 1.506$	1.142	0.255
IQ	$104.292\pm 8.107$	$106.622\pm 9.076$	− 1.849	0.066
SDQ-CP	$7.043\pm 1.628$	$0.256\pm 0.605$	35.040	< 0.001
RPRS Total	$18.661\pm 4.828$	$8.158\pm 2.693$	16.143	< 0.001
RPRS Reactive	$11.016\pm 2.697$	$4.803\pm 2.027$	15.492	< 0.001
RPRS Proactive	$7.683\pm 2.626$	$3.364\pm 0.902$	13.499	< 0.001
Conners (ADHD)	$5.740\pm 6.929$	$0.289\pm 1.008$	7.389	< 0.001

	Percent for CD	Percent for TD		
Male/Female	63/33	59/31	.000 (chi-square)	0.992
MDD	16.7%	0%	4.220	< 0.001
GAD	27.1%	0%	5.751	< 0.001
ADHD	81.3%	0%	19.642	< 0.001
PTSD	16.7%	0%	4.220	< 0.001
CD	100%	0%	–	–
Antipsychotic	11.5%	0%	3.394	< 0.001
Stimulant	20.8%	0%	4.840	< 0.001
SSRI	21.9%	0%	4.993	< 0.001

IQ: Intelligent Quotient; SDQ-CP: Strengths and Difficulties Questionnaire-Conduct Problems; RPRS: Reactive/Proactive Rating Scale; MDD: Major Depressive Disorder; GAD: Generalized Anxiety Disorder; ADHD: Attention Deficit Hyperactivity Disorder; PTSD: Post-Traumatic Stress Disorder; CD: Conduct Disorder; SSRI: Selective Serotonin Reuptake Inhibitor; *p* = two-tailed significance level for the *t* test or chi-squared test

In order to adhere to common clinical practice, diagnoses were determined via detailed interviews with child and adolescent psychiatrists and the participants and parents. The Boys Town National Research Hospital (BTNRH) institutional review board approved this study. A doctoral-level researcher or a member of the clinical research team obtained written informed consent and assent. In all cases, the youth had the right to decline participation at any time before or during the study. With respect to community participants, informed consent was obtained from the youths’ parents/legal guardians at the beginning of the on-site screening. After that, informed assent was obtained from the youth themselves. This procedure differed slightly for youth recruited from the Boys Town campus. Consent was typically obtained from parents during or shortly after the child’s arrival at Boys Town. Assent was obtained from the youth in a separate session, 5–10 days after parental consent had been obtained.

Exclusion criteria included IQ < 75 assessed with the Wechsler Abbreviated Scale of Intelligence (WASI two-subtest form; Wechsler, 2011), pregnancy, non-psychiatric medical conditions that require the use of medication that may have psychotropic effects (e.g., beta blockers or steroids), current psychosis, pervasive developmental disorders, Tourette’s disorder, neurological disorders, presence of metallic objects in the body (e.g., metal plates, pacemakers, etc.), and claustrophobia. Current psychiatric conditions (other than psychotic disorders or pervasive developmental disorders) were not exclusionary. The use of psychotropic medications for psychiatric indications (e.g., stimulants, selective serotonin reuptake inhibitors) was not exclusory. However, participants on stimulant medication were asked to withhold medication on the morning of the scan.

### Data collection

#### Neuroanatomical data

High-resolution structural MRI (T1-weighted) data for each participant was collected using the same 3-Tesla Siemens MRI scanner located at BTNRH. Each participant was instructed to rest, relax, and try their best to minimize head movement during the entire scan. Whole‐brain anatomical data for each participant were acquired using a 3D magnetization‐prepared rapid acquisition gradient echo (MPRAGE) sequence, which consisted of 176 axial slices (slice thickness = 1 mm, voxel resolution = 0.9 × 0.9 × 1 mm^3^, repetition time = 2200 ms; echo time = 2.48 ms; matrix size = 256 × 208; field of view (FOV) = 230 mm, and flip angle = 8°).

#### Measures

Measures included the conduct problems subscale from the Strength and Difficulties Questionnaire (SDQ-CP: [28]), the Reactive/Proactive Rating Scale (RPRS; [29]) to provide information on the severity of aggression in this population, and the Conners ADHD scale [30] to provide information on the severity of ADHD symptomatology in this population. The SDQ-CP shows moderate test–retest reliability [31] and good concurrent validity [32]. Previous research has found evidence supporting the reliability and validity of subscales of the RPRS [33, 34]. The Conners ADHD also had excellent test–retest reliability of 0.89 [35].

### Image preprocessing

The *recon‐all* pipeline from the FreeSurfer toolbox (Version 6.0) was used to process the anatomical brain images [36, 37] and estimate GMV (i.e., CV and SCV). Structural images were processed following the basic image preprocessing steps, *including* head motion‐correction, brain extraction, automated transformation to the standard MNI template space, volumetric segmentation into cortical and subcortical matter, intensity correction, and parcellation of the cerebral cortex into gyral and sulcal matter [38]. For more technical details about the preprocessing steps, interested readers can access previous publications [36, 37, 39]. After preprocessing, we conducted quality control (QC) on each scan using the ENIGMA cortical surface segmentation protocol (for full details and scripts, please see www.enigma.ini.usc.edu). This QC process involved several steps, including outlier detection, visual inspection of surface segmentations for individual scans, and examination of external views of segmentations. Subjects exhibiting structural outliers underwent further scrutiny to address segmentation issues. MATLAB scripts were employed to assess both internal and external surfaces, ensuring the completeness and accuracy of reconstructions, including the presence of all lobes and correct labeling. Subjects were categorized as "pass" (no issues), "moderate" (some regions with potential issues), or "fail" (significant issues). The study initially recruited 1070 participants, with structural MRI data available from 616 participants who successfully passed the QC inspection. Of these 616, a sample of 378 participants (181 were healthy and 197 were diagnosed with CD) were aged ≥ 14 years and had IQ above 84. The final set of participants in healthy (*N* = 90) and CD (*N* = 96) categories were further selected so that the groups matched on age, IQ, and sex—this included the exclusion of (a) participants with disorders (other than CD); (b) participants with highest IQ; (c) youngest controls; (d) participants with lowest IQ; and (e) oldest participants, respectively. This process occurred before the analysis of the sMRI data.

### Data extraction and preparation

The whole brain was parcellated into 68 cortical regions (i.e., 34 regions for each hemisphere) based on Desikan’s atlas [38]. CV data from 68 cortical regions, SCV data from 14 subcortical regions (i.e., 7 regions for each hemisphere), and intracranial volume (ICV; a measure of head size) data were evaluated using the *recon‐all*, *mri_surf2surf*, *mris_anatomical_stats*, and *aparcstats2table* pipelines developed by FreeSurfer.

### Data analysis

#### Clinical data

Group differences in age, IQ, and scores in SDQ-CP, RPRS, and Connor’s ADHD scale were examined via independent samples *t* tests. Group difference in sex was examined via chi-squared tests.

#### Multivariate generalized linear model

The volumes of the right pars opercularis, right pars orbitalis, right pars triangularis, left superior frontal gyrus, right caudal anterior cingulate gyrus, bilateral insula, right superior temporal gyrus, left fusiform gyrus, and left amygdala were used as the dependent variables of the multivariate GLM. The diagnosis of CD was entered as the predictor of the model. ICV, age, sex, and IQ served as the covariates.

### Follow-up analyses

#### Potential confounds

A number of participants with CD were also diagnosed with major depression disorder (MDD; *N* = 16), generalized anxiety disorder (GAD; *N* = 26), posttraumatic stress disorder (PTSD; *N* = 16), and had prescribed medications (e.g., antipsychotic medications, selective serotonin reuptake inhibitors [SSRIs], and/or stimulants; *N* = 37). Our multivariate GLM analysis was therefore repeated four times, once excluding the MDD participants, once excluding the GAD participants, once excluding the PTSD participants, and once excluding the prescribed participants.

#### Whole-brain grey matter volume

To examine if the CD participants and TD participants had different whole-brain GMV, a GLM analysis was conducted on the whole-brain GMV using the diagnosis of CD as the predictor and ICV, age, sex, and IQ as the covariates.

#### Aging effects

In order to address the effects of aging, we formed a new variable by splitting the current CD participants according to the median age and ran a multivariate GLM analysis on the volumes of the pre-hypothesized ROIs by using this new variable as the predictor, and ICV, sex, and IQ as the covariates.

## Results

### Clinical data

As expected, there were no group differences in age, sex, or IQ (*p’*s > 0.05); see Table 1. As expected, participants with CD scored significantly higher than TD participants on the SDQ-CP, the RPRS, and Connor’s ADHD scale; see Table 1.

### Multivariate generalized linear model

**Fig. 1:**
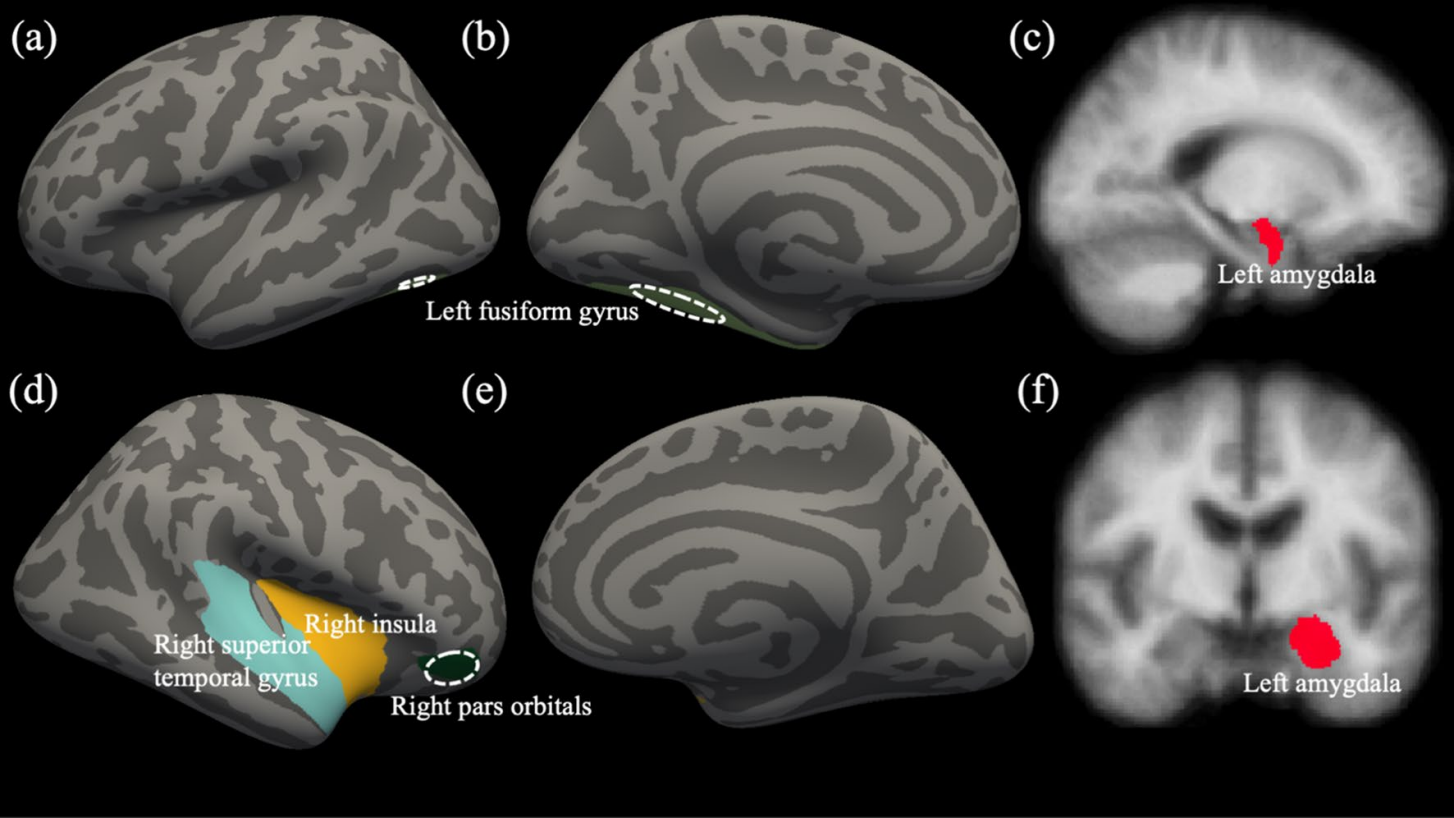
Identified cortical and subcortical regions from multivariate GLM

There was a significant difference in GMV of the pre-hypothesized ROIs based on if the diagnosis of CD was made (*F*_(10, 171)_ = 2.079, *p* = 0.029, ${\eta }_{p}^{2}$ = 0.108, Wilks’ Lambda = 0.892). The diagnosis of CD was a significant predictor for GMV in the right pars orbitalis, right superior temporal gyrus, right insula, left fusiform gyrus, and left amygdala (*F*_(10, 171)_ = 5.460–10.317, *p* < 0.05, ${\eta }_{p}^{2}$ = 0.029–0.054; Fig. 1 & Table 2). Within each of these regions, the CD participants had smaller GMV than the TD participants (M_CD_–M_TD_ = [− 614.898] mm^3^–[− 53.461] mm^3^).

**Table 2:** Multivariate test of diagnosis of CD on volumes of pre-hypothesized regions-of-interests

Region-of-interest^a^	*F* value	*p* value	${\eta }_{p}^{2}$
Left amygdala	5.919	0.016	0.032
Left insula	2.274	0.133	0.012
Right insula	7.334	0.007	0.039
Right pars opercularis	0.024	0.877	0.000
Right pars orbitalis	5.460	0.021	0.029
Right pars triangularis	0.001	0.981	0.000
Right superior temporal gyrus	7.689	0.006	0.041
Left superior frontal gyrus	1.568	0.212	0.009
Right caudal anterior cingulate gyrus	1.299	0.256	0.007
Left fusiform gyrus	10.317	0.002	0.054

^a^According to Desikan’s atlas [38]. ${\eta }_{p}^{2}$ = partial eta squared

### Follow-up analyses

#### Potential confounds

Given the significant associations of CD with other diagnoses and prescribed medications (see Table 1), our multivariate GLM analysis was repeated four times, once excluding the MDD participants (*N* = 16), once excluding the GAD participants (*N* = 26), once excluding the PTSD participants (*N* = 16), and once excluding the prescribed participants (*N* = 37). The four analyses largely replicated the results of our main analysis except that (a) the right pars orbitalis was now trending towards significance when MDD was removed (*p* = 0.057); and (b) the right caudal anterior cingulate gyrus was significant when GAD was removed (*F*_(10, 145)_ = 4.940, *p* = 0.028, ${\eta }_{p}^{2}$ = 0.03; for full details, see online Supplementary Table S1–S4).

#### Whole-brain grey matter volume

To examine if the CD participants and TD participants had different whole-brain GMV, a GLM analysis was conducted on the whole-brain GMV using the same predictor and covariates. The results indicated the CD participants had significantly smaller whole-brain GMV than the TD participants (Mean whole-brain GMV for CD = 590,036 mm^3^, Mean whole-brain GMV for TD = 607,538 mm^3^, *F*_(1, 180)_ = 12.412, *p* < 0.05, ${\eta }_{p}^{2}$ = 0.065).

#### Aging effects

In order to evaluate the aging effects, we divided the current CD participants according to the median age and ran the multivariate GLM analysis on the volumes of the pre-hypothesized ROIs of using this new variable as the predictor, and ICV, sex, and IQ as the covariates. The results indicated there was no significant difference in GMV of the pre-hypothesized ROIs based on if the CD participants were older than the median age or not (*F*_(10, 82)_ = 0.775, *p* = 0.652, ${\eta }_{p}^{2}$ = 0.086, Wilks’ Lambda = 0.914). For full details, see online Supplementary Table S5.

## Discussion

The goal of the current study was to determine the extent to which diagnosis of CD was predicted by atypical volumes within regions identified as atypical in patients with CD in previous meta-analytic work [11]. By implementing multivariate GLM, we demonstrated that diagnosis of CD was predicted by significant differences in volumes of left amygdala, right insula, right pars orbitalis, right superior temporal gyrus, and left fusiform gyrus. In all cases, the participants with CD had smaller GMV than the TD participants. Our results demonstrated the findings of Rogers and De Brito [11] as largely replicable.

The reduction of GMV in the amygdala was consistently demonstrated by earlier CD work [40–45]. Moreover, Rogers and De Brito [11] noted in their review that grey matter reduction in the left amygdala in youths with CP was the most reliable finding that they observed. The current finding of reduced amygdala volume in the CD sample further supports this conclusion. The amygdala is involved in functional processes that have been found to be disrupted in patients with CD including aversive conditioning, decision-making, empathy, and emotional and threat processing [46–51]. Our finding is thus consistent with previous functional MRI data indicating atypical amygdala responding in tasks probing those processes in CD [52–55].

The insula is strongly implicated in empathy and risky decision-making [56]. The role of the insula in empathy has been supported by numerous neuroimaging studies reporting activation in response to others in pain [57]. Its role in risky decisions has been evidenced by robust insular activation in functional MRI studies during gambling tasks, in which the participants had to decide between options associated with uncertain outcomes [58, 59]. Rogers and De Brito [11] found that youths with CP exhibited reduced GMV in the bilateral anterior insula, while the volume of the right insula was reduced in the current CD sample. Reduced volume in the insula has been reported in several CD studies [40, 42, 45] and we replicated these findings. This result aligned with the functional MRI studies reporting altered insula response in youths with CD while watching others with emotional suffering [60, 61] and during decision-making [62]. It suggested that structural abnormality within the insula might partly underlie impaired empathy and poor decision-making in youths with CD.

The superior temporal gyrus has been involved in the perception of facial emotions and social cognition [63–65]. Previous functional MRI studies reported changed activity in the superior temporal gyrus during the processing of emotional faces versus neutral faces in male CD adolescents [54]. In addition, it was reported that the lifetime CD symptoms in female adolescents were negatively associated with activity in the superior temporal gyrus during face processing [66]. The current finding of decreased volume in the superior temporal gyrus in CD aligned with the findings of functional alternation in this region. The current structural finding was consistent with the meta-analysis conducted by Rogers and De Brito [11], although increased volume in the superior temporal gyrus was also reported [13, 67].

CD participants in the current study had smaller right pars orbitalis compared with the TD youth. The pars orbitalis has been shown to be involved in behavioral and motor inhibition and deductive reasoning [68]. The abnormalities within this region may disrupt the internal, intentional cognitive processes of individuals with CD, thereby impeding their ability to anticipate future outcomes of their actions and adhere to socially acceptable behavior.

Further, we observed decreased volume of the left fusiform gyrus in participants with CD. Few previous studies on CD have identified abnormalities in the structural or functional aspects of the visual system. Our discovery in the fusiform gyrus corresponded with the results of a previous study by Jiang et al. [69], which reported a decrease in cortical thickness in the same area in individuals with CD.

Several caveats should be considered with respect to the current results. First, many participants with CD had other psychiatric diagnoses (i.e., MDD, PTSD, and GAD) and/or were prescribed several psychiatric medications (i.e., antipsychotic medications, SSRIs, or stimulants). Ameliorating this concern, the follow-up analyses largely replicated the results of our main analysis. This suggests other psychiatric diagnoses or prescribed medications did not significantly affect the current results. Second, a follow-up GLM analysis revealed that the CD participants exhibited reduced whole-brain GMV compared to the TD participants. However, the primary research findings included ICV as one of the covariates. As such, our primary research findings could not be attributed to head size/total GMV differences between groups. Third, according to a follow-up multivariate GLM analysis, we observed no significant difference in GMV of the pre-hypothesized ROIs based on if the CD participants were older than the median age or not; i.e., within this sample, older CD participants did not show reduced GMV relative to younger CD participants—in contrast to some previous studies suggesting that higher CD-related symptomatology was associated with accelerated age-related cortical alteration. Fourth, diagnostic status was determined following clinical practice and included an interview by a board-certified psychiatrist rather than the implementation of a structured or semi-structured diagnostic interview. While the diagnosis method we used could raise concerns regarding the CD diagnoses, it is important to note that these diagnoses were supported by the SDQ-CP scores.

In conclusion, we found that volumes of the left amygdala, right insula, right pars orbitalis, right superior temporal gyrus, and left fusiform gyrus were reduced in the CD participants relative to the TD participants. These regions may serve as biomarkers for the development of CD in adolescents. Clinical work can potentially target those biomarkers for future therapeutic interventions as well as predict patients’ disease trajectories.

### Supplementary Information

Below is the link to the electronic supplementary material.

Supplementary file1 (DOCX 28 KB)

## Data Availability

The data that support the findings of this study are available from the corresponding author upon reasonable request. The data are not publicly available due to IRB restrictions.
