# Supplementary material for: Reduced grey matter volume in adolescents with conduct disorder: a region-of-interest analysis using multivariate generalized linear modeling
Source: Discov Ment Health. 2023 Nov 17;3(1):25. doi: 10.1007/s44192-023-00052-3 (PMC10656392; doi:10.1007/s44192-023-00052-3)
Supplement: Supplementary file 1 — Supplementary file1 (DOCX 28 KB) [file 44192_2023_52_MOESM1_ESM.docx]

Table S1. Multivariate test of diagnosis of CD on volumes of pre-hypothesized regions-of-interest, CD participants with MDD removed.

| **Region-of-interest**^a^ | ***F* value** | ***p* value** | $\boldsymbol{\eta}_{\boldsymbol{p}}^{\boldsymbol{2}}$ |
| --- | --- | --- | --- |
|  |  |  |  |
| Left amygdala | 5.578 | 0.019 | 0.033 |
| Left insula | 1.479 | 0.226 | 0.009 |
| Right insula | 8.000 | 0.005 | 0.047 |
| Right pars opercularis | 0.177 | 0.675 | 0.001 |
| Right pars orbitalis | 3.680 | 0.057 | 0.022 |
| Right pars triangularis | 0.080 | 0.778 | 0.000 |
| Right superior temporal gyrus | 7.460 | 0.007 | 0.044 |
| Left superior frontal gyrus | 0.822 | 0.366 | 0.005 |
| Right caudal anterior cingulate gyrus | 1.376 | 0.243 | 0.008 |
| Left fusiform gyrus | 8.794 | 0.003 | 0.051 |
|  | | | |

Key to Table S1: ^a^According to Desikan’s atlas (Desikan et al., 2006). $\eta_{p}^{2}$ = partial eta squared.

Table S2. Multivariate test of diagnosis of CD on volumes of pre-hypothesized regions-of-interest, CD participants with GAD removed.

| **Region-of-interest**^a^ | ***F* value** | ***p* value** | $\boldsymbol{\eta}_{\boldsymbol{p}}^{\boldsymbol{2}}$ |
| --- | --- | --- | --- |
|  |  |  |  |
| Left amygdala | 4.834 | 0.029 | 0.030 |
| Left insula | 2.272 | 0.134 | 0.015 |
| Right insula | 8.773 | 0.004 | 0.054 |
| Right pars opercularis | 0.084 | 0.773 | 0.001 |
| Right pars orbitalis | 9.662 | 0.002 | 0.059 |
| Right pars triangularis | 0.151 | 0.698 | 0.001 |
| Right superior temporal gyrus | 9.776 | 0.002 | 0.060 |
| Left superior frontal gyrus | 2.872 | 0.092 | 0.018 |
| Right caudal anterior cingulate gyrus | 4.940 | 0.028 | 0.031 |
| Left fusiform gyrus | 10.529 | 0.001 | 0.064 |
|  | | | |

Key to Table S2: ^a^According to Desikan’s atlas (Desikan et al., 2006). $\eta_{p}^{2}$ = partial eta squared.

Table S3. Multivariate test of diagnosis of CD on volumes of pre-hypothesized regions-of-interest, CD participants with PTSD removed.

| **Region-of-interest**^a^ | ***F* value** | ***p* value** | $\boldsymbol{\eta}_{\boldsymbol{p}}^{\boldsymbol{2}}$ |
| --- | --- | --- | --- |
|  |  |  |  |
| Left amygdala | 8.721 | 0.004 | 0.050 |
| Left insula | 2.891 | 0.091 | 0.017 |
| Right insula | 8.764 | 0.004 | 0.051 |
| Right pars opercularis | 0.017 | 0.896 | 0.000 |
| Right pars orbitalis | 6.910 | 0.009 | 0.040 |
| Right pars triangularis | 0.197 | 0.658 | 0.001 |
| Right superior temporal gyrus | 7.785 | 0.006 | 0.045 |
| Left superior frontal gyrus | 1.215 | 0.272 | 0.007 |
| Right caudal anterior cingulate gyrus | 3.674 | 0.057 | 0.022 |
| Left fusiform gyrus | 8.596 | 0.004 | 0.050 |
|  | | | |

Key to Table S3: ^a^According to Desikan’s atlas (Desikan et al., 2006). $\eta_{p}^{2}$ = partial eta squared.

Table S4. Multivariate test of diagnosis of CD on volumes of pre-hypothesized regions-of-interest, CD participants with prescription removed.

| **Region-of-interest**^a^ | ***F* value** | ***p* value** | $\boldsymbol{\eta}_{\boldsymbol{p}}^{\boldsymbol{2}}$ |
| --- | --- | --- | --- |
|  |  |  |  |
| Left amygdala | 5.357 | 0.022 | 0.036 |
| Left insula | 0.209 | 0.648 | 0.001 |
| Right insula | 3.967 | 0.048 | 0.027 |
| Right pars opercularis | 0.021 | 0.886 | 0.000 |
| Right pars orbitalis | 5.426 | 0.021 | 0.037 |
| Right pars triangularis | 0.190 | 0.664 | 0.059 |
| Right superior temporal gyrus | 9.011 | 0.003 | 0.006 |
| Left superior frontal gyrus | 0.885 | 0.348 | 0.029 |
| Right caudal anterior cingulate gyrus | 1.880 | 0.173 | 0.013 |
| Left fusiform gyrus | 7.340 | 0.008 | 0.049 |
|  | | | |

Key to Table S4: ^a^According to Desikan’s atlas (Desikan et al., 2006). $\eta_{p}^{2}$ = partial eta squared.

Table S5. Multivariate test on volumes of pre-hypothesized regions-of-interest based on if the CD participants were older than the median age or not.

| **Region-of-interest**^a^ | ***F* value** | ***p* value** | $\boldsymbol{\eta}_{\boldsymbol{p}}^{\boldsymbol{2}}$ |
| --- | --- | --- | --- |
|  |  |  |  |
| Left amygdala | 0.801 | 0.373 | 0.009 |
| Left insula | 2.523 | 0.116 | 0.027 |
| Right insula | 0.056 | 0.813 | 0.001 |
| Right pars opercularis | 1.613 | 0.207 | 0.017 |
| Right pars orbitalis | 0.143 | 0.706 | 0.002 |
| Right pars triangularis | 1.483 | 0.227 | 0.016 |
| Right superior temporal gyrus | 1.144 | 0.288 | 0.012 |
| Left superior frontal gyrus | 0.249 | 0.619 | 0.003 |
| Right caudal anterior cingulate gyrus | 0.209 | 0.648 | 0.002 |
| Left fusiform gyrus | 0.533 | 0.467 | 0.006 |
|  | | | |

Key to Table S5: ^a^According to Desikan’s atlas (Desikan et al., 2006). $\eta_{p}^{2}$ = partial eta squared.

**References**

Desikan, R. S., Ségonne, F., Fischl, B., Quinn, B. T., Dickerson, B. C., Blacker, D., . . . Killiany, R. J. (2006). An automated labeling system for subdividing the human cerebral cortex on MRI scans into gyral based regions of interest. *Neuroimage, 31*(3), 968-980. doi:10.1016/j.neuroimage.2006.01.021
